# Supplementary material for: The relationship between chronic health conditions and cognitive deficits in children, adolescents, and young adults with down syndrome: A systematic review
Source: PLoS One. 2020 Sep 11;15(9):e0239040. doi: 10.1371/journal.pone.0239040 (PMC7485757; doi:10.1371/journal.pone.0239040)
Supplement: S2 Table — (DOCX) [file pone.0239040.s002.docx]

**Table S2.1: Domains of the Cochrane Risk of Bias in Systematic Reviews (ROBIS)**

| **Domain 1: Study Eligibility Criteria** | |
| --- | --- |
| *Did the review adhere to predefined objectives and eligibility criteria?* | Yes |
| *Were the eligibility criteria appropriate for the review questions?* | Yes |
| *Were eligibility criteria unambiguous?* | Yes |
| *Were all restrictions in eligibility criteria based on study characteristics appropriate?* | Yes |
| *Were any restrictions in eligibility criteria based on source of information appropriate?* | Yes |
| *Concerns regarding specifications of study eligibility:* Low  *Rationale for concern:* No concerns  *Describe research criteria:* The inclusion and exclusion criteria were prespecified. Articles were limited to publications reporting on both chronic health conditions and associated cognitive performance in individuals with Down syndrome. | |
| **Domain 2: Identification and Selection of Studies** | |
| *Did the search include an appropriate range of databases/electronic sources for published and unpublished reports?* | Yes |
| *Were methods additional to database searching used to identify relevant reports?* | Yes |
| *Were the terms and structure of the search strategy likely to retrieve as many eligible studies as possible?* | Yes |
| *Were restrictions base on date, publication format, or language appropriate?* | Yes |
| *Were efforts made to minimize error in selection of studies?* | Yes |
| *Concerns regarding methods used to identify or select studies:* Low  *Rationale for concern:* No concerns  *Described methods*: An academic medical librarian extracted articles from two large publication databases using Medical Subject Heading (MESH) terms involving Down syndrome, specified chronic health conditions, and cognition. | |
| **Domain 3: Data Collection and Study Appraisal** | |
| Were efforts made to minimize error in data collection? | Yes |
| Were sufficient study characteristics available for both review authors and readers to be able to interpret the goals? | Yes |
| Were all relevant study results collected for use in the synthesis? | Yes |
| Was risk of bias formally assessed using appropriate criteria? | Yes |
| Were efforts made to minimize error in risk of bias assessment? | Yes |
| *Concerns regarding methods used to collect data and appraise study:* Low  *Rationale for concerns:* No concerns  *Describe methods:* Data were extracted from two large publication databases into an excel document and two independent reviewers rated the inclusion criteria initial based on titles and abstracts, and subsequently based on the full articles. Risk of bias was assessed using the Cochran ROBIS tool. | |
| **Domain 4: Synthesis and Findings** | |
| *Did the synthesis include all studies that it should?* | Yes |
| *Were all predefined analyses reported or departures explained?* | Yes |
| *Was the synthesis appropriate given the nature and similarity in the research questions, study designs, and outcomes across included studies?* | Yes |
| *Was between-study variation minimal or addressed in the synthesis?* | Yes |
| *Were the findings robust as demonstrated through funnel or sensitivity analysis?* | No |
| *Were biases in primary studies minimal or addressed in the synthesis?* | Yes |
| *Concerns regarding the synthesis and finds:* Low  *Rationale for concerns:* No concerns.  *Describe synthesis method:* The synthesis was appropriate given the nature and similarity in the research questions, study designs, and reported outcomes across studies included in the synthesis. Biases were low across all articles included in the review synthesis. | |

**Table S2.2: Overall risk of bias in the review based on the Cochrane Risk of Bias in Systematic Reviews (ROBIS)**

| **Overall Risk of Bias in the Review** | |
| --- | --- |
| *Did the interpretation of findings address all of the concerns identified in Domains 1- 4?* | Yes |
| *Was the relevance of identified studies to the review's research question appropriately considered?* | Yes |
| *Did the reviews avoid emphasizing results on the bases of their statistical significance?* | Yes |
| *Risk of bias in the review:* Low  *Rationale for risk:* Low concerns were identified in regard to the study eligibility criteria, identification and selection of studies, data collection and study appraisal, and synthesis and findings. The conclusions of the review were supported by evidence of associations between cognitive abilities and specific chronic health conditions in individuals with Down syndrome. | |
